# Evaluating the impact of a network of research partnerships: a longitudinal multiple case study protocol

**DOI:** 10.1186/s12961-018-0377-y

**Published:** 2018-11-12

**Authors:** Femke Hoekstra, Kathleen A. Martin Ginis, Veronica Allan, Anita Kothari, Heather L. Gainforth

**Affiliations:** 1 School of Health and Exercise Sciences, University of British Columbia Okanagan, Kelowna, BC Canada; 2 School of Kinesiology and Health Studies, Queen’s University, Kingston, ON Canada; 3 School of Health Studies, Western University, London, ON Canada; 4 International Collaboration on Repair Discoveries (ICORD), University of British Columbia, Vancouver, BC Canada; 5 Department of Medicine, University of British Columbia Okanagan, Kelowna, BC Canada

**Keywords:** Research partnership, Integrated knowledge translation, Stakeholder engagement, Research co-production, Participatory research, Mixed-methods, Impact assessment, People with physical disabilities, practitioners, Health system leaders

## Abstract

**Background:**

Conducting and/or disseminating research together with community stakeholders (e.g. policy-makers, practitioners, community organisations, patients) is a promising approach to generating relevant and impactful research. However, creating strong and successful partnerships between researchers and stakeholders is complex. Thus far, an in-depth understanding of how, when and why these research partnerships are successful is lacking. The aim of this study is to evaluate and explain the outcomes and impacts of a national network of researchers and community stakeholders over time in order to gain a better understanding of how, when and why research partnerships are successful (or not).

**Methods:**

This longitudinal multiple case study will use data from the Canadian Disability Participation Project, a large national network of researchers and community stakeholders working together to enhance community participation among people with physical disabilities. To maximise the impact of research conducted within the Canadian Disability Participation Project network, researchers are supported in developing and implementing knowledge translation plans. The components of the RE-AIM framework (reach, effectiveness, adoption, implementation and maintenance) will guide this study. Data will be collected from different perspectives (researchers, stakeholders) using different methods (logs, surveys, timeline interviews) at different time points during the years 2018–2021. A combination of data analysis methods, including network analysis and cluster analysis, will be used to study the RE-AIM components. Qualitative data will be used to supplement the findings and further understand the variation in the RE-AIM components over time and across groups.

**Discussion:**

The outcomes, impacts and processes of conducting and disseminating research together with community stakeholders will be extensively studied. The longitudinal design of this study will provide a unique opportunity to examine research partnerships over time and understand the underlying processes using a variety of innovative research methods (e.g. network analyses, timeline interviews). This study will contribute to opening the ‘black box’ of doing successful and impactful health research in partnership with community stakeholders.

**Trial registration:**

Open Science Framework: https://osf.io/kj5xa/.

## Background

The process of transferring research findings to practice and policy is complex and often unsuccessful [1–4]. If research findings are not translated, community stakeholders (e.g. policy-makers, practitioners, community organisations, patients) cannot benefit from the best available knowledge and healthcare. As a result, research cannot have an impact in society. Moreover, lack of adequate translation results in a large amount of research waste in terms of the invested time and money to conduct the research [5, 6]. One of the reasons for this ‘knowledge-to-practice gap’ is that the needs and research priorities of researchers do not always correspond with the needs and priorities of those who may benefit from the research [7, 8]. Engaging stakeholders in the research process has been proposed as a promising approach to close this ‘knowledge-to-practice gap’ [9–12]. An example of such an approach is integrated knowledge translation (KT), in which researchers and stakeholders work collaboratively in the research process [9]. The extent to which stakeholders are involved in all phases of the research process can vary from project to project. Independent of the extent of involvement, it requires a strong and successful partnership between the academic researcher(s) and the community (i.e. ‘research partnership’) [13].

Over the last decades, research partnerships have become increasingly popular, as illustrated by the large number of literature reviews published on different types of research partnership approaches (cf. [11, 14–19]). Across the fields of health and social sciences, many research partnerships have been created to conduct research together on a broader network level (e.g. [20–22]) as well as on an individual project level (e.g. [23–25]). Although conducting research within a research partnership is popular, setting up a partnership does not happen spontaneously. Partnership formation is often a complex and lengthy process. In addition, setting up a sustainable partnership is even more challenging [26]. To date, many studies, including literature reviews, have focused on identifying hampering and facilitating factors to creating successful research partnerships (cf. [11, 15, 16]). Commonly mentioned barriers include excessive time investment, excessive funding pressures, unclear roles and/or functions, and poor communication between members of the partnership [15]. On the other hand, commonly mentioned facilitators include trust, respect and a good relationship among partnership members, shared vision and/or goals, and effective communication [15]. However, an overview of important hampering and facilitating factors is not enough to understand how, when, with whom and why partnerships are successful (or not) in conducting and/or disseminating research. Understanding these underlying processes is important to provide effective support and guidance to researchers and stakeholders on working collaboratively, which may subsequently contribute to more relevant and impactful research [10, 27].

Nonetheless, studying the underlying processes of a successful partnership is challenging due to its multi-factorial character and its context-dependent successes. Despite its complexity, several studies have been conducted to start opening this ‘black box’ of successful research partnerships (cf. [14–16, 28–30]). To date, most of the studies were cross-sectional, using only qualitative methods [15]. No studies have been found that measure research partnership quality and synergy over time. Moreover, the majority of the studies included only one case (i.e. one partnership), instead of multiple cases. Partnerships and projects are heterogeneous, in itself illustrating the necessity to study multiple cases. Given their common goals and processes, networks in which researchers and community stakeholders are working together on different projects and in different partnerships are an ideal setting to study the underlying processes of partnerships and to understand their heterogeneity [20–22].

### Canadian Disability Participation Project (CDPP)

The CDPP network is meant to enhance community participation among Canadians with physical disabilities [31]. The research projects within the CDPP network focus both on improving and understanding the quantity (i.e. the number of people who participate) as well as the quality of participation (i.e. the quality of peoples’ participation experiences and satisfaction [32]). The network aims to enhance quantity and quality participation in three areas, namely employment, mobility, and sport and exercise. To date, the CDPP network consists of 31 principal researchers and 18 community stakeholders (organisations).

Within this network, researchers and stakeholders work in partnerships to conduct and/or disseminate their research with community stakeholders following an integrated KT approach. A well-trained KT specialist supports researchers in developing and implementing plans to translate their research findings to practice and policy, aiming to maximise the impact of CDPP research. The goal of this service is to build KT capacity among researchers. Although the CDPP partnerships are all KT focused, the degree to which stakeholders are engaged in the research process varies from project to project. Moreover, while the research projects all focus on participation, they differ in their research scope, topic and design. The heterogeneity in partnership characteristics provides an opportunity to gain a better understanding of how, when, with whom and why research partnerships are successful in conducting and/or disseminating research together, and how this is related to impact. These insights may contribute to the development of more effective and sustainable research partnerships, and may help to further optimise the support offered to researchers and stakeholders regarding how to conduct and/or disseminate research together.

### Guiding framework

This study will use the RE-AIM framework as a guide to understand the outcomes and impacts of the CDPP at the network and project levels [33]. This framework includes the following five components: Reach, Effectiveness, Adoption, Implementation and Maintenance. The RE-AIM framework was originally developed to study the public health impact of interventions and programmes. In this study, we elaborated on the work of Sweet et al. [34], who illustrated how the RE-AIM framework can be used as a guide to evaluate the impact of a large partnership between researchers and community stakeholders. We will operationalise and measure the RE-AIM components at different levels (network and project) and at different moments in time. Moreover, we will explain the variation in the RE-AIM components (over time and among partnerships) to gain a better understanding of how to conduct successful and impactful research within a research partnership.

### Study aims

The aim of this study is to evaluate and explain the outcomes and impacts of the CDPP at the broader network level, as well as at the level of individual projects, using the RE-AIM framework. In doing so, this project will offer an enhanced understanding of how, when, with whom and why research partnerships are successful (or not). More specifically, this study aims to identify how the RE-AIM components change and vary over time at the network and project levels. Ultimately, this study will contribute to further opening the ‘black box’ of doing successful and impactful research in partnership with community stakeholders.

## Methods

### Study overview

**Fig. 1:**
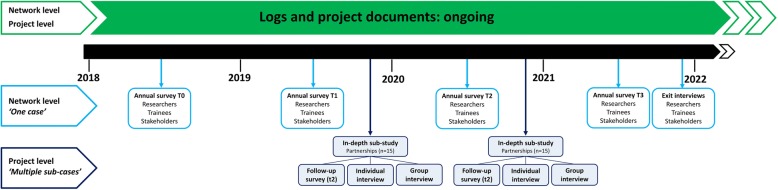
An overview of the data sources and moments of measurement at the network and project level

An overview of the study is depicted in Fig. 1. A two-level longitudinal multiple case study design using quantitative (logs, surveys) and qualitative research methods (exit interviews, timeline interviews) will be used. Data will be collected from different perspectives (researchers and stakeholders), and at different time points between the years 2018 and 2021. The two-level design consists of (1) the CDPP network (‘one case’) and (2) the projects of research partnerships within the CDPP network (‘multiple cases’).

### Study population

At the network level, the study population consists of all researchers, research trainees and community stakeholders (e.g. decision-makers, patients, community organisations) that are affiliated with the CDPP network during the study period (2018–2021). To be included, participants (researchers, research trainees and community stakeholders) need to be 18 years and older, and give consent to participate in this study.

At the project level, the study population consists of researchers, research trainees and community stakeholders involved in one or more CDPP research projects during the study period (2018–2021). CDPP research projects are all projects initiated by one of the principal researchers of the CDPP network and (partly) funded by CDPP. Inclusion criteria for researchers, research trainees and community stakeholders are (1) being actively involved in one or more CDPP research projects, (2) 18 years and older, and (3) giving consent to participate in this study. For the in-depth sub-study (Fig. 1), a selection of partnerships working on a CDPP research project will be invited. Only CDPP projects that are in the dissemination stage (i.e. have results to be disseminated) will be included. We will use a maximum variation sampling [35] method to recruit partnerships that vary the most with regards to the following characteristics: (1) partnership quality and synergy, (2) partnership size, (3) geographical spread, and (4) research topic and area. Since researchers, research trainees and community stakeholders can be part of different partnerships, we will allow them to participate a maximum of two times in the in-depth sub-study if they belong to more than one partnership. We will recruit partnerships until data saturation is achieved.

### RE-AIM components

Table 1 presents a complete description and operationalisation of the five RE-AIM components used to guide the study at the network and project levels.

**Table 1:** Operationalisation of the RE-AIM components at network and project levels

RE-AIM component	General description	Operationalisation	Data source
Reach			
Network	The amount and type of information that is sent out to the community from the CDPP network yearly	Total number of academic papers, academic presentations, community presentations, non-academic papers, policy briefs and reports, articles, blogs and papers on websites, social media posts, mass media press releases, guides, toolkits, videos and aids (KT products) yearly	Logs + project documents
Project	The amount and type of information that is sent out to the community by each CDPP research project	Mean, ranges and frequencies of academic papers, academic presentations, community presentations, non-academic papers, policy briefs and reports, articles, blogs and papers on websites, social media posts, mass media press releases, guides, toolkits, videos and aids (KT products) per project	Logs + project documents
Effectiveness			
Network	Multidirectional flow of knowledge: an increase in the multidirectional flow of knowledge across disciplines and sectors within the CDPP network over time	Changes in the network analysis measures (e.g. density, reciprocity, core-periphery structure) of the CDPP network over time	Logs + project documents, exit interviews
	Researchers’ capacity for KT activities: an increase in researchers’ capacity for KT activities before and after the support from the KT consultation service, and an increase over the time of the study period (2018–2021)	Changes in researchers’ capability, opportunity and motivation to translate their research findings to a non-academic audience before and after they received support from CDPP’s KT consultation service Changes in researchers’ capability, opportunity and motivation to translate their research findings to a non-academic audience over time (2018–2021)	Project surveys, annual surveys, exit interviews
Project	Partnership quality and synergy: an optimisation of partnership quality and synergy in the process of conducting and/or disseminating research in a research partnership	Identification of partnership profiles based on indicators of partnership quality and synergy Changes in indicators of partnership quality and synergy over time	Project surveys, annual surveys, timeline interviews
Adoption			
Network	The extent to which CDPP researchers decide to contact the KT consultation service to translate their research findings to an academic and non-academic audience	Percentage of CDPP researchers that contacted the KT consultation service Number of times that CDPP researchers have consulted KT consultation service The total number and duration of the support provided by the KT consultation service	Logs + project documents
Project	The extent to which CDPP research projects are conducted and disseminated in a research partnership	Percentage of CDPP research projects that are conducted in partnership with community stakeholders Percentage of CDPP research projects that are disseminated in partnership with community stakeholders Percentage of CDPP research projects that are conducted and disseminated in partnership with community stakeholders Percentage of CDPP research projects that are not conducted or disseminated in partnership with community stakeholders	Logs + project documents
Implementation			
Network	The extent to which goals stated by the directors and team leads of the CDPP are achieved (i.e. implementation as intended)	Conformity of intended network’s goals and achieved goals	Logs, project documents, exit interviews
Project	The extent to which the CDPP partnerships have conducted and/or disseminated their research according to the KT plan (i.e. implementation as intended)	Conformity of intended KT plan and KT evaluation	Logs, project documents
Maintenance			
Network	A sustainable multidirectional flow of knowledge across disciplines and sectors within the CDPP network		Annual survey (T3), exit interviews
	The long-term capacity among CDPP researchers for KT activities		Annual survey (T3), exit interviews
Project	The continuation of conducting and/or disseminating research projects in a research partnership (i.e. ‘sustainable partnerships’)		Project surveys, timelines interviews

*CDPP* Canadian Disability Participation Project, *KT* knowledge translation

### Reach

At the network level, ‘reach’ refers to the amount (e.g. number of presentations) and type of information (e.g. scientific versus public) that is sent out from the CDPP network to the community. Changes in the amount and type of information sent out by the network will be measured to assess how the ‘reach’ of the network will change over time. At the project level, ‘reach’ refers to the amount and type of information sent out by each CDPP research project. Throughout the study period, trained research coordinators will be collecting this information (e.g. number of presentations, papers, KT products) via research reporting forms and up-to-date CVs of the involved CDPP researchers. In addition, administrative project documents, like a KT plan and project evaluations, which the principal researchers need to provide for each project, will be used. Descriptive statistics (e.g. means, percentages, frequencies) will be used to describe the change over time and differences across sectors, disciplines and projects.

### Effectiveness

At the network level, the effectiveness component focuses on an increase in (1) the multidirectional flow of knowledge and (2) researchers’ capacity for KT activities.

#### Multidirectional flow of knowledge

The changes in the multidirectional flow of knowledge over time (2018–2021), across disciplines (mobility, employment, sport and exercise) and sectors (e.g. academic, community, policy) will be evaluated using network analysis [36, 37]. A network analysis is a mathematical and graphical method of analysing complex, interpersonal processes and can be used to understand how knowledge flows within networks or organisations. Conducting network analyses will provide an opportunity to visualise the CDPP network structure over time using sociograms and describe the network’s characteristics using several measures at both the network (e.g. density, core-periphery structure) as well as the individual level (e.g. degree, closeness) [38–41]. Logs and project documents will be used to collect information about the researchers, trainees and stakeholders (contact persons and organisations) involved in each of the CDPP research projects. Each year of the study period, a network analysis will be conducted and key network level measures will be determined. This analysis will allow us to identify how the CDPP network is changing over time in terms of memberships and collaborations.

To understand and explain how the CDPP network is functioning and changing over time, a selection of the CDPP researchers and community stakeholders will be invited for an ‘exit interview’ at the end of the study period (Fig. 1). The exit interviews will focus on researchers’ and stakeholders’ experiences with the CDPP network, their view on how the CDPP network has changed the multidirectional flow of knowledge, and their view on how the CDPP network has influenced the KT capacity among researchers, as well as related facilitators and barriers. A key element of the exit interviews will be to discuss and reflect on changes in the CDPP network structure over time (2018–2021) [42]. To guide this discussion, the interviewer will show the interviewees the sociograms generated through the network analyses. After explaining these sociograms, the interviewer will ask the researcher or stakeholder to reflect on changes in the sociograms over time. Moreover, the interviewer will ask questions about researchers’ or stakeholders’ own position within the CDPP network and how that position has changed over time. Probing questions will be asked to gain more insight into how, when and why their position within the CDPP network structure has changed or remained stable, in addition to perceived facilitators and barriers.

Examples of interview questions and prompts include (1) “Please tell me about your experiences being a part of the CDPP network”, (2) “What are, in your opinion, successes of the CDPP network?”, (3) “What are, in your opinion, challenges of the CDPP network?”, (4) “How has the CDPP network structure changed over time, and how do you explain these changes?”, (5) “How do you feel about your position within the CDPP network?”, and (6) “What lessons did you learn from being part of the CDPP network?”

Furthermore, all members of the CDPP network will be asked to complete an online survey yearly (T0: August 2018, T1: August 2019; T2: August 2020: T3: August 2021) in order to obtain information about their general demographics, their experiences with the CDPP network, the value of the CDPP network, and the quality of the communication within the network, as well as questions about researchers’ capacity for KT activities. The survey will be adapted to the participant’s role in the network (researcher or stakeholder). Filling out the survey will take approximately 10 min.

Quantitative data collected with annual surveys will be described and tested with appropriate statistics using IBM SPSS Statistics for Windows Version 24.0. All exit interviews will be audio recorded and transcribed verbatim. Qualitative data will be analysed thematically to explore trends and patterns in participants’ experiences with and perceptions of the CDPP network [43]. The analysis will be conducted using the software programme NVivo 11 for Windows.

#### Researchers’ capacity for KT activities

Changes in researchers’ capacity for KT activities will be assessed before and after receiving support from the KT specialist and will be assessed over the study period (2018–2021). Researchers’ capacity for KT activities will be measured using behaviour change theory and will be specifically focused on researchers’ capability, opportunity and motivation (i.e. COM-B [44]) to translate their research findings to a non-academic audience. According to the COM-B model, researchers must have perceived capability, opportunity and motivation to engage in a certain behaviour [44]. To examine changes in researchers’ capability, opportunity and motivation for translating research findings to a non-academic audience, researchers will be asked to fill out a short survey at three moments in time, namely pre-survey (t0), post-survey (t1) and follow-up survey (t2). The pre- and post-surveys will, respectively, be conducted immediately before and after the support from the KT specialist, whereas the follow-up survey (t2) will be conducted 1 year later. Furthermore, items related to researchers’ capacity for KT activities will be included in the annual surveys (T0–T3).

Qualitative data from the ‘exit interviews’ will be used to supplement and explain the findings of the survey data about researchers’ capacity for KT activities using triangulation [35]. In addition, we will use the information about the form, frequency and content of the actual support provided by the KT specialist to explain and understand if researchers’ KT capacity has been changed over time. The KT specialist will therefore register details about the support by logging the form (internet, phone, face-to-face) and duration of the meetings with the researchers. In addition, every meeting will be audio recorded to collect details about the content of the meetings.

At the project level, effectiveness refers to an optimisation of partnership quality and synergy in the process of conducting and/or disseminating research in a research partnership (Table 1). Partnership quality is operationalised using nine indicators for a successful partnership identified by Kothari et al. [45], namely communication, collaborative research, dissemination of research, research findings, negotiation, partnership enhancement, information needs, level of rapport, and commitment. Partnership synergy refers to the extent to which the partnership combines the complementary knowledge, skills, and resources of all members of the partnership to create new ideas and look for better ways to solve problems and achieve goals [46–48]. The idea is that, by creating synergy the partnership can achieve more than any of its individual members and become “*a whole that is greater than the sum of its parts*” [48]. Partnership synergy is considered a key indicator for a successful collaboration [47, 48]. Insight into different partnership profiles of indicators and partnership quality and synergy will help to gain a better understanding how, when, with whom and why research partnerships are successful.

**Fig. 2:**
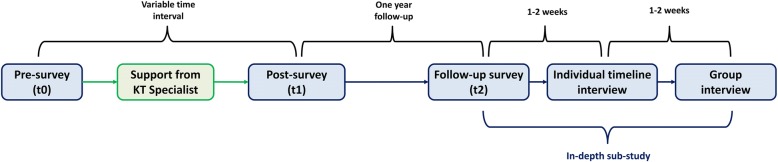
An overview of the data sources and moments of measurement at the project level

To evaluate the effectiveness component at the project level, a selection of research partnerships will be studied in-depth (Figs. 1 and 2*)*. All key members of the selected partnerships (researchers, trainees, stakeholders) will be invited for an additional in-depth sub-study including the follow-up survey (t2) and two timeline interviews (Fig. 2). A timeline interview is a qualitative research tool that can be used to study individuals’ experiences over time [49]. More specifically, it is a way to link personal stories or narratives with a broader context. In this study, timeline interviews will be used to obtain information about researchers’, trainees’ and community stakeholders’ experiences working together on a predetermined CDPP research project. We expect the number of participants per partnership will vary from two to five, depending on the size of each partnership. The in-depth sub-study consists of three parts, namely the follow-up survey, an individual timeline interview and a group interview (Fig. 2).

#### Part I: follow-up survey

All members of the selected partnership will be asked to fill out the follow-up survey (t2) to obtain information about partnership quality and synergy from a researcher and stakeholder perspective. Partnership quality will be assessed using an adapted version of the Partnership Indicators Questionnaire [21, 45]. Because the Partnership Indicators Questionnaire was originally developed in the context of policy-maker–researcher partnerships, we adapted it for our sample by piloting the questionnaire with researchers and stakeholders who had experience with working together on research projects.

Partnership synergy will be assessed using the nine items of the Partnership Self-Assessment Tool [47, 48]. A partnership synergy score will be calculated reflecting the extent to which the members of a partnership are achieving more together than they can on their own. Completing the survey (pre-survey, post-survey, follow-up survey) will take approximately 10 min per survey. The follow-up survey (part I) will motivate researchers, trainees and stakeholders to think about their partnership prior to the individual timeline interview (part II). In addition, this survey will help the interviewer to prepare the timeline interviews and identify possible probing questions.

#### Part II: individual timeline interview

Second, all members of the selected partnerships will be invited for an individual timeline interview. During this interview, the interviewer and the interviewee will co-create a timeline of the interviewee’s experiences with conducting and/or disseminating research in a research partnership, from the first communication about working together on the research project to present. The timeline will mark key moments and activities throughout the research process, such as ‘first meeting with partners’, ‘decision to collaborate’, ‘discussing research plan’, ‘ethical procedures’, ‘ethical approval’, ‘start of data collection’, ‘start of data analysis’, and ‘discussion of research findings’. The interviewee will lead the process of adding key moments and activities to the timeline and, if necessary, the interviewer will ask the interviewee for permission to include other generic milestones that typically occur throughout the research process (e.g. defining the research question). For each moment or activity included on the timeline (i.e. moment in the research process), the interviewer will ask questions such as: (1) “How did you feel during that moment?”, (2) “Describe the relationship between you and your partners?”, (3) “On a scale of 1 to 10, how would you rate your general satisfaction level regarding the partnership functioning?”, (4) “What barriers did you perceive at this moment?”, and (5) “What facilitators did you perceive at this moment?”. Because the interviewer and interviewee will work together to develop the timeline, the interviewee will have an active role in the reporting process. As such, the interviewer and interviewee will have shared ownership and analytic power during the interview session [49]. All interviews will be conducted by the first author (FH) in person or online using a video conversion interface (Vidyo). If interviews are conducted in person, the timeline will be created on paper. If interviews are conducted via internet, the timeline will be created using an online application. The interviewer may take additional notes during the interview sessions. After each session, the created timelines will be shared and verified with the participants.

#### Part III: group interview

Third, all key members of the partnership will be invited for an interview session together, allowing the interviewees the opportunity to interact with one another. Prior to this group interview session, the interviewer will combine the individual timelines and experiences of all members of the partnership into one group timeline. During the group session, all members of the partnership will be asked to discuss and reflect on this group timeline. By doing so, the experiences of all partnership members will be compared and differences and similarities will be discussed. The interviewer will ask questions such as (1) “Can you reflect on this moment or activity?”, (2) “What went well?”, (3) “What did not go as expected?”, (4) “What would you do differently if you could do it over?”, (5) “How did your relationship differ between different moments on the timeline?”, and (6) “In general, what lessons did you learn from this partnership?”

The last part of this group interview will focus on general indicators for a research partnership. The interviewer will ask questions such as (1) “In your opinion, what is a successful research partnership?” and (2) “How should researchers and community stakeholders ideally work together?”. The interview questions will be piloted and the interview guides will be adjusted accordingly.

#### Data analysis

All interview sessions will be audio recorded and transcribed verbatim. The transcripts, notes and created timelines will be analysed using an inductive thematic analysis approach to explore trends and patterns in participants’ experiences with working in partnership on a CDPP research project [43]. All qualitative data collected at the project level (individual timeline interviews and group interviews) will be analysed collectively. The analysis will be conducted following the six steps described by Braun et al. [43], which include familiarisation with the data, coding of the data, developing the themes, refining the themes, naming the themes and writing up the findings. We will illustrate the themes with quotations from participants. The analysis will be conducted using the software programme NVivo 11 for Windows. We will use a flexible approach to determine the appropriate and relevant criteria to evaluate the quality of our qualitative research methods [50, 51].

Following our inductive analysis of the qualitative data, we will conduct a mixed method analysis of the quantitative and qualitative data (Parts I–III) to inform a better understanding of how, when, with whom and why research partnerships are successful. The mixed method approach will be conducted in two main steps. First, we will conduct a hierarchical cluster analysis [52] to identify different profiles of partnerships based on indicators of partnership quality and synergy measured at the follow-up survey (t2). Each partnership will be grouped into a *k* number of profiles (i.e. clusters). Within each of these profiles, partnerships will be most similar to each other in terms of partnership quality and synergy, but they will be most different from other profiles (i.e. minimum within-profile variation and maximum between-profile variation). Second, we will determine factors and characteristics of research partnerships that are associated with the identified partnership profiles. Both quantitative survey data (annual surveys, pre- and post-surveys) and qualitative data (timeline interviews) will be used to describe the partnership profiles and determine factors and characteristics associated with successful partnerships. For this analysis step, the qualitative data will be re-analysed using a deductive approach focusing on specific factors and partnership characteristics. The identified partnership profiles will help us gain a further understanding of how, when, with whom and why research partnerships are successful (or not) by explaining the variation between partnerships and by gaining insights on how partnerships are functioning over time.

### Adoption

At the network level, adoption describes the extent to which CDPP researchers decide to contact the KT consultation service to translate their research findings to academic and non-academic audiences (Table 1). At the project level, adoption describes the extent to which CDPP research projects are conducted and disseminated in a research partnership. The logs and project documentation will be used to assess the adoption outcome at the network and project level.

### Implementation

At the network level, implementation focuses on the extent to which the goals stated by the directors and team leads of the CDPP network are achieved at the end of the study period (i.e. implementation as intended). At the project level, implementation focuses on the extent to which the CDPP partnerships have disseminated their research findings according to their KT plan. Therefore, the planned KT activities mentioned in the KT plan will be compared with the KT activities that are actually completed as reported in the project documents.

### Maintenance

At the network level, maintenance refers to a sustainable multidirectional flow of knowledge across disciplines and sectors within the CDPP network and the long-term capacity for KT activities among CDPP researchers. Information about the maintenance of the CDPP network will be obtained using the annual survey T3 and the exit interviews (Fig. 2). At the project level, maintenance refers to the continuation of conducting and/or disseminating research projects in a research partnership (i.e. sustainable partnerships). Information about researchers’ and stakeholders’ views on the continuation of their partnership will be collected during the timeline interviews (Fig. 2).

## Discussion

This paper outlines a protocol to study the outcomes, impacts and processes of conducting and disseminating research together with community stakeholders at different levels (network and project), using the RE-AIM framework, in the context of research among people with physical disabilities. The longitudinal design of this study will provide a unique opportunity to study research partnerships over time and to understand the underlying processes using a variety of innovative research methods (e.g. network analyses over time, timeline interviews).

This study will contribute to the science of KT, implementation, partnerships and impact evaluation. By using the RE-AIM framework as a guide to study the impact of a network of research partnerships, we elaborated on the work of Sweet et al. [34]. Using the RE-AIM framework, we will be able to collect, organise and interpret our data in a structural and systematic way. As such, we will have the capacity to build a rich dataset, thereby increasing the knowledge base regarding the creation of effective and sustainable research partnerships. In this way, we hope to further open the ‘black box’ of successfully conducting and disseminating research in partnership with stakeholders.

In addition to these scientific contributions, this study will also have practical contributions. The findings of this study may be used by many groups working in the areas of health, social sciences and beyond. First, researchers who work or want to work together in partnership with community stakeholders may benefit from the findings of this study. The project level data will provide new insights into the underlying processes of conducting and/or disseminating research in partnership. These insights may guide researchers in making decisions regarding how, when and with whom stakeholders should be engaged in their research processes. Moreover, the identified partnership profiles of partnership quality and synergy may be used to optimise the tailored support for researchers on how to work in a research partnership. More specifically, we will use the findings of our study to improve and expand the CDPP’s KT support services to further enhance KT capacity among researchers. Ultimately, our findings may be used by researchers from all levels as a guide to create and sustain research partnerships, which may substantially contribute to closing the ‘knowledge-to-practice gap’ within health and social science research.

Second, community stakeholders who are or want to be involved in conducting and/or disseminating research may benefit from the findings of this study. Stakeholders with different backgrounds, experiences, roles and responsibilities will participate in our study allowing us to learn from this heterogeneity and gain a better understanding of what works for whom the best. The insights may help stakeholders in decision-making processes regarding their engagement in research processes on both a network and project level. We expect that, if stakeholders have a better overview of the potential costs and benefits of working together on a research project, more efficient and effective research partnerships can be created. Furthermore, we hope that our network level data will show the value and impact of a large KT-focused network of researchers and community stakeholders, such as the CDPP, which may inspire researchers and stakeholders in other fields and other countries to set up similar (inter)national networks.

Lastly, funding agencies of partnered research may benefit from the findings of this study. For example, in Canada, partnered research approaches are becoming increasingly incentivised as evidenced by the granting opportunities across different federal agencies that require partnership building (e.g. Social Sciences and Humanities Research Council of Canada Partnership Development and Partnership Grants). Our findings may be used as a first step in developing criteria for creating successful research partnerships in health and social sciences. Such criteria may help funding agencies decide how to spend their money. Having such criteria will also help to monitor and evaluate the funded partnered research projects. Furthermore, we are currently reviewing and synthesising the literature on research partnerships in collaboration with researchers from different institutions across Canada [53]. In the future, we hope that the findings of this literature overview, in combination with the findings of the current study, will be used to develop guidelines for conducting and disseminating research together with community stakeholders. Such guidelines will not only help funding agencies, but also researchers and stakeholders working together in partnership.

In summary, this study will gain a better understanding of the underlying processes of research partnerships working together on research projects to enhance participation among people with physical disabilities. Therefore, the findings of this study may be relevant for a broad audience, including health researchers, community stakeholders and funding agencies. Moreover, this study will contribute to opening the ‘black box’ of doing successful and impactful health research in partnership with community stakeholders.

## Abbreviations

CDPP: Canadian Disability Participation Project
KT: knowledge translation
